# Ninth International Medical Congress, Washington, D. C., Sept., 1887

**Published:** 1887-12

**Authors:** 


### PROCEEDINGS.

### Ninth International Medical Congress, Washington, D. C., September, 1887.

SECTION XVIII. DENTAL AND ORAL SURGERY.

(Reported by Mrs. M. W. J. for Dental Register.)

(*Continued from page* 564.)

THURSDAY, Sept. 8, 11 A. M. The first paper read was by Dr. L. C. Ingersoll (Keokuk, Iowa).

Owing to the large number of papers still to be read, Dr. Ingersoll stated that he would only read selections from his paper on

INFLAMMATORY PROCESSES, wrongly announced on the programme as "Inflammation in the Oral Tissues."

He said that he would not attempt to go into the etiology nor the bacteriology of inflammation, its pathology being the most important to us, as the source of much human suffering which we might possibly relieve. The course of inflammation was illustrated by a chart, as follows:

| | | | | | |
|---|---|---|---|---|---|
| Irritation. | Inflammation acute. | Congestion. | Necrosis. | Exfoliation. | Somatic death. |
| | | | Gangrene. | Slough. | |
| | | | Suppuration. | Molecular death. | |
| | | Inflammation chronic. | Hypertrophy. Tumefaction. Induration. | | |

In inflammation there is always something in the environment of the parts not in harmony, and which causes deviations from the physiological to the abnormal—a retrograde metamorphosis. In all nature there are dual forces in operation, building up or disintegrating. As in electricity there is positive and negative, so the positive and negative in physics are physiology and pathology.

Passing over the portion of the paper devoted to irritation, Dr. Ingersoll continued: Should irritation continue, the vessels become permanently dilated and molecular changes occur, bringing us to the second stage, or acute inflammation. We might call it hyperæmia, fluxion, determination, but our patients—the common people—call it inflammation. The results of continued inflammation depend upon constitutional diathesis and the severity of the injury. It may terminate in necrosis, gangrene, or suppuration, or it may become chronic and result in hypertrophy, tumefaction, or induration of the tissues. In an exposed tooth pulp a breath of air, a particle of food, or a drop of water may cause excruciating pain, but by the use of palliative remedies and a training to new habits it will learn to tolerate so much that the patient may think it dead.

We may expect congestion after acute inflammation; it is the shortest, quickest route to the death of the tissues. Chronic inflammation is the "main line;" hyperæmia is a "stop-over station." In hyperæmia the tissues are overfed, more blood is supplied than is needed, and it is utilized in the production of more tissue than is needed, though it is impossible to see where the new growth begins. In tumefaction the elevation differs in color and in texture, with a strawberry or cauliflower look. The excrescence arises from normal tissue as the result of inflammation; the excess of blood is utilized in a new line, a perversion of function producing a new kind of tissue. In hyperæmia normal tissue is abnormally excited; in tumefaction abnormal tissue is produced by normal function. Tumors have some of the characteristics of their own tissues, but a tumor of the pulp is painless as compared with the healthy pulp. They sometimes acquire an enormous size compared with the normal pulp, more than filling the crown cavity.

Passing over induration, Dr. Ingersoll went back to acute inflammation followed by congestion. Vascular action is lessened, the blood current is retarded, and corpuscles are massed, clogging up the canals, causing blood stasis. This is accompanied by a throbbing pain, which is the pathological characteristic of congestion. If blood stasis is complete, gangrene follows at once. The formation of pus has been the source of more discussion than any other feature of inflammation. In suppuration there is molecular death, dying by particles. The diseased parts are not wholly dead, but they are throwing off dead particles, migratory, embryonic cells, blood corpuscles, micro-organisms, dead cells. In blood stasis nutrition is diminished, the vascular tissues are weakened, function is impaired. Unless it is resolved disastrous results must follow. Red corpuscles are massed, white corpuscles float slowly along and percolate into the neighboring tissues; changing shape, they pass through the walls of the vessels, red corpuscles following. But nature has her reconstructive forces everywhere at work. When the corpuscles break through the walls they meet broods of embryonic tissue cells at work on repairs. But for this work of construction in the midst of destruction speedy death would supervene. Alveolar abscess would mean not merely swelling and pain but the death stroke. Passing to necrosis, inflammation of enamel may occur in the reticulum of living matter, causing atrophy. In inflammation of cancellous bone—as of the maxillaries about the apex of the root of a tooth—there is no redness or swelling, but the medullary tissue may be truly inflamed, at the expense of the mineral walls, which are softened and broken down. What was called inflamed dentine twenty-five years ago is now more generally termed sensitive dentine; but when dentine is keenly sensitive it is evidence of inflammation of the fibrils which form a plexus of vital tissue, not sensitive in normal condition. A pulp exposed by fracture may be touched with the finger or instruments without pain. Inflammation of the pulp may be caused by the irritation of the fibrils by a metal filling two lines distant from the pulp-chamber. This fact establishes the fact of the continuity of living matter throughout the tooth. *Pulp* means an inert homogeneous structure. The nerve center of the tooth should be called the dental ganglion. We might as well call the brain the cranial pulp as to speak of a dental pulp! The correct representation of the tooth-pulp, since we must use the term, should show the fibrils radiating in all directions—a central organ with radiating processes. The process of inflammation in dentine is analogous to that of bone. The medullary portion expands at the expense of the mineral walls of the tubuli, having so-called softened dentine. Inflammatory action is an important factor in diseases of tooth structure.

Dr. A. O. Rawls (Lexington, Ky.) opened the discussion of this paper. He said he would not attempt to add any thing to, or to gainsay any thing contained in the paper relating to the pathology of inflammation. It was all stated in many books by many authors, whom Dr. Ingersoll had followed quite closely. He did not think Dr. Ingersoll's position could be maintained when he differed from Conheim and Stricker, as in the combined work of leucocytes and embryonic tissue cells. He thought Conheim correct in the statement that pus was due strictly to the death by strangulation of leucocytes in passing through the walls. He also thought Dr. Ingersoll's drawing of a pulp tumor rather that of a fungous growth due to strangulation. He could not agree with Dr. Ingersoll that pus was living tissue; it was simply broken-down tissue. Leucocytes are living wandering cells, but when they pass out by ameboid movement they have lost their place in nature and are no more living. He could not conceive of healthy granulations over carious surfaces, and he also believed that sensitive dentine was the result of a hyperæmic condition of the contents of the tubuli, caused by packing in an additional amount from the increased flow of blood to the parts; a congested condition, but not due to inherent sensitiveness. If an exposed pulp is compressed it is always painful, but as the result of compression, not from inherent sensitiveness. The disease of dentine spoken of by Dr. Ingersoll he did not think the result of inflammation in general, but of that special form of inflamma tion, congestion, causing absorption and breaking down. Dentine melts away under compression, not because of special inflammation, but because of the rapid flow of the watery part of the blood to the dentine.

Dr. Fletcher (Cincinnati) had made some interesting experiments in electricity in this connection. Isolating the mesentery of a frog, leucocytes gathered along the edges of the capillaries till it was completely occluded. When migration began the battery was connected and the Farradic current turned on. At once the course of the migratory leucocytes was checked and they died *en route*, strangled by the way, and circulation was re-established. This would seem to show that there was something in the life of the corpuscles that was affected by electricity, and other irritating applications.

Dr. Storey (Texas) said that all his theories were getting upset. He had been taught that in inflammation there was stasis; the blood was dammed up and could not get away. But as he understood the paper, the new teaching was that there was an increased and more rapid flow of blood. He had also been taught that pus, as pus could never enter into the circulation of the blood; poisonous matters could enter; bacteria and microbes might be found in the circulation, but pus never.

Dr. Ingersoll said that the diagram shows not only progression but retrogradation. By lancing or other treatment at certain stages, we carry it back to acute inflammation again when it may follow another track; instead of reaching induration or tumufaction, it may go on to suppuration, etc. The coating of the capillary vessels consists of cells glued together. Comparison might be made with a cup of flaxseed and a little water, the migration of corpuscles being like insects passing out between the seeds. There are no *stomata* but they may pass out anywhere.

If we irritate the ends of the fibrils, by continuity of tissue we irritate the pulp, but the dentine is not irritated.

Dr. M. L. Rhein (New York) thought Dr. Rawls erroneous in antagonizing Dr. Ingersoll as to the possibility of healthy granulation over carious portions of bone. The sequestrum is encysted and there is proliferation of new tissue cells and there is thus healthy granulation around carious portions. He thought Dr. Rawls was eminently right as to Dr. Ingersoll's illustration of pulp tumor, which was hypertrophy.

Dr. Ingersoll said that, having read only scattered portions of his paper, he had not made himself clearly understood on some points. He hoped they would read it all in print. They would never call that hypertrophy if they accepted the definition of the best authors.

Dr. C. H. Goddard (San Francisco) read a paper entitled—

### Pain in the Temporo-Maxillary Joints, Caused by Irregularity of the Teeth.

This was the history of a case in which the patient complained of severe pain in mastication. It was at first thought to be due to an impacted wisdom tooth, but on examination they were found to be all in place and with plenty of room. There was no tenderness on pressure, anywhere, and the cause of the pain, which was so severe that he could not masticate a crust of bread, was not apparent. Examinatian of the articulation showed that the inferior maxillary was longer than the other, and the incisors met on the cutting edge, the bicuspids and molars not antagonizing by one-sixteenth of an inch. To bring them together in mastication it was necessary to protrude the inferior maxillary unnaturally. It was apparent that the pain was due to the long continued strain of the muscles and ligaments in an unnatural position. The question then was what change in articulation would be best; whether to shorten the inferior maxillary or bring the superior arch forward? The occupation of the patient, a young man of thirty years of age, was such as to prevent his wearing any apparatus that would show or interfere with his voice as he was teaching singing to young ladies. A Coffin split-plate was adopted, which he wore comfortably, and in a few weeks the superior incisors closed outside of the inferior, and a normal articulation was secured, and in this simple way, a long standing pain, or disease, was cured.

As the hour for adjournment was at hand, the next paper was read by title, subject:

Influence of Weather Changes on the Human Organism, by Dr. E. S. Chisholm (Tuscaloosa, Ala).

Dr. Chisholm said he had hoped to have an opportunity to read this paper, as a long series of observations covering over fifteen years had convinced him that the subject opened up a broad field for scientific investigation in which he wanted the dental section to lead the way. It was a subject that he felt was worthy the consideration of the International Medical Congress, and of governmental cooperation and investigation. When we reflect that there is an atmospheric pressure of fifteen pounds to every square inch of surface, we can conceive that changes in the character of the atmosphere and of barometric pressure must have an effect upon the human body—its nerves and tissues. From the results of his observations he had learned to feel certain, from the state of his pocket barometer in the morning, the class of patients, or rather suffering, that he would have to deal with through the day. He would only allude to this and give the title of his paper.

The President, Dr. Taft, said that at the suggestion of Dr. Chisholm, he had been making similar observations for six or eight years, and was much impressed with the importance of the subject, and its practical bearing professionally, and he regretted that there was not time to read the paper.

Adjourned.

Thursday, September 8, 3 P. M.

Dr. W. W. Allport (Chicago) in the chair.

Dr. E. S. Talbot (Chicago) read a paper entitled

### Etiology of Irregularities of the Jaws and Teeth.

Dr. Talbot observes that irregularities of the jaws and teeth are more frequent now than among earlier peoples or lower grades of humanity. Seeking for the causes of these irregularities, we first note that the maxillary bones grow and develop quite independently of each other; that the inferior jaw which has greater mobility is supplied, in conjunction with the fixed superior maxillary, with blood, vessels, etc., the separate bones developing simultaneously but independently of each other until ossification of the sections. From insufficient blood supply or other causes there may be arrested development, and consequent irregularity. The increasing irregularities of the present races is due also to the comparative inaction of the maxillaries. In place of the roots and nuts and coarse grains which required laborious mastication we have soft foods ready for deglutition when they enter the mouth. The anatomist speaks of the inferior and superior maxillæ, but the inferior maxilla is really two separate bones, united at the mental foramen, while above we have the jawbone, the palatal bones. When the teeth erupt there is a new growth called the alveolar process, which is absorbed when the deciduous teeth are shed, and developed again with the permanent teeth, the bony maxilla continuing its growth and development independently of the process, the latter depending entirely on the teeth for its growth and shape. If the teeth come through the inner border, when dentition is complete the diameter is less than that of the jawbone. If they come through on the outer border, then the diameter is greater. Eruptive fever, or other constitutional disturbances in children, may cause an arrest of development in the maxillary bones. This is also characteristic of idiots, imbeciles, and feeble-minded children; with but few exceptions the congenital idiot has the arch contracted in width between the bicuspids.

Dr. Talbot then gave some very valuable statistics and deductions from examinations of the teeth in a large number of instiutions for the above classes, including those at Syracuse, N. Y.; Boston; Randall's Island, N. Y.; Minnesota; Kansas; Pennsylvania; the Cook County Insane Asylum, and others. Out of 1605 examined 924 had normal mouths, 87 had very large jaws or teeth, 63 were prognathous lower jaw, 97 ditto upper, 238 high arch, 101 V-shaped, 200 partial V-shaped, 31 thumb-suckers, 159 saddle arch, 56 abnormally small teeth. The examinations were made among all grades from the absolute idiot to the merely feeble-minded, and in all social grades. Endeavors were also made to ascertain whether the irregularity was due to external causes, as lack of attention to the deciduous teeth, thumb-sucking, etc. The percentage of known thumb-suckers was very small. As the result of arrested development two very distinct forms are recognized—the V-shaped arch and the saddle-shaped. The V-shaped arch is always associated with the superior maxillary, and is never found with the deciduous teeth; the central incisors are turned with the mesial surface towards the lips, the laterals are turned posteriorly towards the median line, the cuspids are outside of the arch, the bicuspids and first molars dropping forward, and all the teeth crowded; it is always associated with enlarged tonsils and mouth-breathing. Dr. Kingsley thinks it is inherited and congenital; Dr. Oakley Coles attributes it to premature ossification of the sutures; Dr. Cartwright to lax habits of life in ancestry; many dentists attribute it to thumb-sucking. Dr. Ballard (England) considers thumb-sucking the forerunner of idiocy. The alveolar process is long and thin, the teeth are very long and wedge-shaped, the cuspids have very long roots and being outside of the arch have great leverage. The laterals are very small and crowded forward and rotated with the cutting edge forward. The tongue is contracted and elongated, and in its turn shapes the contour of the process; the hard palate may be either low or vaulted.

The saddle-shaped arch is usually formed in the superior maxillary, but sometimes in the inferior. Here the central incisors protrude vertically with the alveolar process and close together. The alveolar process is on the inner border of the bone, to the bicuspids and first molars, and outside for the second and third molars. The cuspids are either placed normally or outside. The cuspids and first molars occupy a fixed point, the bicuspids are crowded inwardly. The first molars and the second bicuspid form an inclined plane and are turned in their sockets. The cuspids erupt before the bicuspids, and the arch is high and vaulted.

It is universally recognized that various morbid conditions are transmitted, though they may be either enhanced or lessened in succeeding generations—sometimes entirely disappearing when prescribed by environment. Malformed individual teeth are transmitted with great regularity. In one case a mother, daughters, and grand daughters, had fissured lateral incisors. One child may be like the father, and another like the mother; one may transmit a peculiarity of the maxillary and another of the teeth. Much has been said as to the effect of ante-natal influences on the development of the teeth and jaws, but evidence on this head is difficult to obtain. In one instance a mother was very much worried lest her child should have an irregularity like her own. It proved to be identical. Here there was probably both heredity and ante-natal influence; it is evident that the same influence acting in the other direction might prevent heredity from acting. Local causes probably produce the mâjority of cases of irregularity otherwise the children ought to be just like the parents. In a certain family the father's jaw was well developed with large strong teeth ; the mother's teeth were small and regular in the inferior maxillary, but the superior centrals and laterals had been extracted at the age of seven years leaving the cuspids in position. The son's teeth were very irregular—like the father's in quality, but their position was inherited from the mother. At fourteen years of age the laterals were far inside, and the cuspids, bicuspids, and molars all anterior to their natural position. A younger son has inherited the same tendency, the cuspids encroaching on the laterals and tending to produce the V-shaped arch, showing the transmission of shape of jaws and peculiarity of individual teeth.

Dr. Mummery, in the Odontological Society of Great Britain, reports from his researches in 3,000 skulls his conclusion that the early and half savage peoples were freer from irregularities than modern people.

There is never any irregularities in the teeth of the Chinese. The nomadic races also have perfectly developed teeth or arches. In new countries irregularities may result from intermarriage of different races—the crossing of racial peculiarities. The abuse of the teeth is due to depraved hygiene—not to civilization. The nearer the monkey, the further from man, the better the teeth ; the less we depend upon them, the less perfect they become.

Dr. Talbot concluded that thumb-sucking rarely produces irregularity of the pernanent teeth. The effects on the teeth, if any, are neither uniform nor extensive. If very persistent it may prevent the eruption of the deciduous teeth, or the development of the alveolar process ; the inferior maxillary may be crowded back, the arch may be changed to an oval shape. The habit of thumb-sucking is contracted in infancy while the teeth are imperfectly developed and may be easily modified by the pressure of the thumb, lips, and tongue. The habit is, however, usually outgrown by the time the permanent teeth erupt, and the malformed alveolar process of the deciduous teeth being absorbed, the permanent teeth will assume their proper shape.

If not, their fan-shaped protrusion is marked and peculiar; the inferior maxillary crowded inward, with spaces between the teeth of the superior maxillary; the arch and the hard palate, however, are not changed.

The regular proceedings were here suspended in honor of the presence of the venerable Dr. N. S. Davis, President of the Congress.

In introducing Dr. Davis to the members of the section, Dr. Taft said:

The members of the XVIII Section of the International Congress do not need to be told that the dental profession occupies a very different position to-day from that which it held four months ago. Our success of to-day is dependent in a goodly measure on action taken at that time. Embarrassments have been removed which then seemed insurmountable. The influence of this action reaches not only the men here to-day, but it reaches the profession throughout the world wherever dentistry is practiced. The one to whom we are indebted for this is present with us to-day--the President of the IX International Medical Congress has been the friend of dentistry for a quarter of a century past, and he has done more than any man in the medical profession for the profession of our choice.

Dr. Taft then introduced Dr. N. S. Davis as the friend of the dentist and of dentistry. When the applause had subsided Dr. Davis said, in substance, that he had no thought of interrupting the order of business, but that he had thought it a part of his duty to look through all the sections that he might judge for himself of the success of the congress. He congratulated the XVIII Section on its large attendance and earnest work. He said that he had long felt the dental branch of medicine should not differ from the other departments; that all were aiming at the same goal, all striving to reach a reliable scientific plane, which could only be done through harmonious action. The day had gone by for founding sects and schools on theoretical dogmas. Medicine, to be worthy of its name, should be founded on scientific investigation of accumulated facts, drawing conclusions by inductive logic. He said, in 1865, when your organization met in Chicago,

I invited some of your members to a simple entertainment in my own house. Being called upon to say something, I said that our teeth, our jaws, were as important portions of the human organization as the eye, the ear, or any other organ. The knowledge of how to treat their diseases, accidents and injuries, requires the same scientific knowledge of the principles of medicine as the treatment of any other organ. I then predicted that dentistry, opthalmology, otology, or any other *ology* would eventually stand on the same plane—the great broad field of medicine. The twenty-two years that have passed have seen dental schools develop. Dental departments have been organized in our best universities, where there are medical departments with the same professors and the same classes, with degrees granted by the same authorities. It seemed in the last medical association that the time had come when the dental branch should no longer stand alone. Your educational demands are the same, except that you require *more* in your concentrated clinical study. It only needed the official declaration on the line that whoever practices dentistry on the authority of that education shall stand on the same level, shall be recognized as a part of the medical profession as far as national organizations go. I did not misjudge. There was not a dissenting voice. It was adopted officially. The educated part of the dental profession is now part and parcel of the broad field of medicine. As co-laborers I hail you, gentlemen, in that capacity. If I live a few years more I hope to see disappear the last vestige of the pretended schools of medicine. There are no "schools" in the proper sense of the term. Homœopath, Eclectic, Allopath—let all come on to the broad platform as doctors of medicine, without any *pathy*. Let us labor to that end—no schools, no *pathies*, no *isms*, but one broad field of science, and that alone. [Tremendous Applause.]

Dr. Taft: I am very sure that no one here will ever forget the grand and cheering words we have just heard. It makes us feel better to have been so greeted.

Prof. Busch, of the dental department of the University of Berlin, the representative of the dental profession in Germany (who had just arrived), was next introduced.

Speaking in German, he expressed his regrets at having been detained by a rough passage of fifteen days, crossing the Atlantic. He was, however, delighted to have arrived in time to have heard the kind and encouraging address of the President of the Congress. He had been struck and gratified by the words "brother dentists" —on placing the dentists emphatically on the same plane as his medical brethren. It has been his endeavor to reach the same position in Germany, but they had not yet attained that equality of title, though the studies were the same—more stringent than in America, especially in preliminary education. He said that he had come to America to see and examine the American system and to post himself as to the educational methods practiced in this country.

Business was again resumed, and Dr. Edw. H. Angle (Minneapolis) read a paper entitled:

### Orthodontia, with a New System of Regulation and Retention.

Dr. Angle said that the principles called into play in all cases of regulating were few, and consisted merely in moving the teeth either forward or backward in the line of the arch—from within or from outside the arch into line, or in elongating or depressing individual teeth. Only one tooth should be moved at a time, otherwise the liability of inflammation is increased. The movement may be constant or intermittent, fast or slow, as judgment dictates, but no retrogression must be permitted. No one mechanical power is of so universal application as the screw; it is powerful, compact, and not unsightly. The next most important force is the spring of piano wire. Removal of the apparatus by slipping or breaking or for cleansing is the most frequent cause of failures and also of strangulation and death of the pulp. The appliance should be such that there will be no occasion for removal until the design is accomplished. The resistance should be sufficient to overcome the resistance of the tooth to be moved, but should never impinge upon the gum; neither should the crown of a tooth be covered by plates of vulcanite or metal, interfering with occlusion. Far too little importance is attached to the retaining appliance. The teeth should be firmly supported and retained in place until perfectly firm in their new position, and no movement allowed to disturb or interfere with the formation of new bone. The teeth should be held as securely as a fractured limb in a splint, and for the same reasons. The alveolar growth will not be healthy under unfavorable conditions, as in case of inflamed gums, or mal-occlusion, or a removable apparatus. For all these reasons ligatures and plates are not the best means of regulating. We want simplicity and cleanliness. The best method is delicate bands of platinum or gold cemented to the teeth to be moved. A strip of 32 to 36 gauge platinum, one-eighth inch in width, can be passed around the tooth, the ends grasped with flat-nosed pliers and burnished to the form of the tooth. The ends can then be passed through a piece of hollow joint-wire and cemented to the tooth. When all the teeth to be moved are thus "banded and piped" a band of platinized gold is cut to the required length and adjusted to the arch, the teeth that are to be moved from within outwards being pushed with a jack-screw; those from without drawn in by a traction-screw, rotation being effected by an application of piano-wire with an eye at one end. In all cases the rubber-dam should be put on before the teeth are banded.

Dr. A. W. Freeman asked how he would prevent the posterior tooth of resistance from moving forward?

Dr. Angle replied that as many teeth could be banded and piped as was thought necessary to afford ample resistance.

(Owing to the absence of the Secretary, Dr. J. J. R. Patrick, Belleville, Ill., was unable to read his paper on "Irregularities," as per programme.)

The discussion of the subject was opened by Dr. J. N. Farrar (New York). He said that the ground gone over was so extensive that it would be impossible to review it thoroughly. Dr. Talbot's paper alone would require ten hours for proper discussion. He could not entirely agree with him as to his *first causes*, but that was only a matter of opinion; there was not yet enough known on the subject. He considered the observations in asylums as very important, though he had been very much dissatisfied with the results of his own observations in that field. We often think we know a thing, but when we trace it out more fully we find that our conclusions were all wrong. To formulate any truly scientific deductions we should have the type of the parents for at least two generations. The results of the mixing of types and nationalities are pretty well established. Along the border lines of nations of different origin there is more irregularity than in nations that have been preserved from such contact. It is generally supposed there is correlation between the prognathus jaw and the sloping forehead, but there may be good intelligence with prognathus jaw if the forehead is upright; if the forehead is sloping the individual is apt to be odd and peculiar if not weak. There is a blending along the line of idiocy and insanity, but one needs more facts. There is great discrepancy between the observations made in the British Islands and in this country, either there has been lack of care, or the observers could not see the pointing fingers, through what they saw.

As to the second paper, mechanical appliances used in regulaing teeth act upon different principles. He did not approve of an appliance which should operate with continued force, beyond the control of the patient. It should act intermittently, and at will, in the judgment of the patient short of actual pain. The movement should be within the domain of physiological action and the highest possibilities. The tissue changes in the socket are not necessarily pathological, and need not involve soreness and pain which are pathological in character. Retrograde metamorphosis, if kept within certain limits, is painless, while if carried beyond a certain point it is painful and no longer physiological.

Pain may be sometimes justifiable, to economize time and expense, but to the extent that it is avoidable pain is in violation of the law of harmony, though not evidence of malpractice. The interests of humanity, however, demand that regulation shall be without pain when circumstances permit, though they are sometimes such as to render it impossible to avoid it. The most scientific method is "the best under the circumstances." When the roots are only partially developed the alveolar tissues are soft and with little or no sensitiveness, and the teeth can be moved quickly and with little or no pain. If the apparatus is not removable filthiness is an almost invariable concomitant. The tissues will more readily tolerate intermittent force, and the physiological principle is always the most scientific. The system of Dr. Angle is not *a new system* as will be recognized by all journal readers. All the appliances that he uses can be found described in the journals. Dr. Farrar said that he was placed in a delicate position in this matter as it was known that he had a large work on the subject just coming out. Anyone, however, who will to go over the *Cosmos, the Independent Practitioner, the* REGISTER, *Missouri Journal,* will see for themselves that Dr. Angle's system is not new.

Dr. Haskell (Chicago) said that Dr. Angle's pictures (charts illustrating his paper) failed to give a correct idea of his appliances, which struck him as being admirable. He did not pretend that the Jackscrew *per se* was new, but his were so exceedimgly small that they were really entirely different from anything he (Dr. Haskell) had ever seen. In his " system " he uses no plates, no rubber bands, no ligatures, and it is all exceedingly simple and very easily applied. A set of his appliances, and some of his plaster casts would have been much better than his charts. I do not know that any one has observed one fact that I have learned from my work on several hundred plates for the upper jaw, which is that there is always a marked depression on the left side, requiring the teeth to be set longer than on the right side ; they also often require to be set farther in, or thrown out of the arch on the left side in arranging a set of teeth. What is the cause of this ; it antedates extraction. Is it from the same cause that makes people right-handed—that when they want to bite a piece off of any thing, they hold it in the right hand?

Dr. Gish (Michigan) reported the case of a lady of twenty-five years of age whose teeth had been regulated, correcting a tush-like projection of the incisors. The irregularity was easily corrected in three months. She wore a retaining plate for three months longer when the teeth seemed to be perfectly tight, in a beautiful arch. At the end of a week the incisors were found to be working back to their original position, and she wore the retaining plate four weeks longer, but within twenty-four hours after removal the teeth began to move back again. She then wore the retaining plate for a year, but with no better result.

Dr. Allport (Chicago) said that the present points were not in order. The discussion should be confined to the subject of the papers read.

Dr. Wm. N. Morrison (St. Louis) said that Dr. Angle was not entitled to credit for the annular bands. At the Cincinnati meeting of the American Dental Association he (Dr. Morrison) had shown their use for attaching screws, etc., to regulating appliances. At the clinics he would show an apparatus which would correct the most difficult case in twenty-one days.

Dr. W. C. Barrett urged that this was all foreign to the true objects of the session which was to discuss principles, not appliances, or precedents, or claims.

To his apprehension, in Dr. Talbot's paper one force had not been alluded to which is potent in the production of peculiarities in the jaw, and that is simply the occlusion of the teeth after the eruption of the permanent teeth. He would simply throw out the suggestion, not desiring to occupy time. Mal-occlusion of the teeth is the frequent cause of mal-formation of the jaws, though the influence of heredity is a very potent factor. It has been said that the nearer we get back to the primeval type the more perfect the jaws and teeth. We have been presented with the statistics of 4,000 prehistoric skulls, which show that as near as we can judge the type of teeth and jaw has not materially altered. The so-called "rudimentary condition" of the wisdom teeth has not changed in three thousand years in the prehistoric races of the old world. Back of the white people here we find no difference, except as the result of diseases developed through contact with the whites. There were no teeth of the syphilitic type previous to the advent of the white people. As to the progress of caries, it is the same to-day as three thousand years ago, in nearly the the same proportion and virulence. The ravages of disease were as prevalent then as now. He could not conceive of any material change in the type of teeth, of diseases of the oral cavity, of caries of the teeth, or of the type of jaw. Different races of men have different types of teeth, and the type of jaw forms a national characteristic. While we have three national types we have also individual peculiarities in the perpetuation of which heredity is potent, while mal-occlusion is potent as a factor in their causation. He (Dr. Barrett) therefore takes exception to the statement as not being a matter of record that the farther back we go the more perfect the type, except as lowered by the intermingling of races.

Dr. I. A. Salmon (Boston): One practical point that he had discovered in his practice which has not been mentioned is the advantage of bringing a tooth outward *beyond* the point required. Even when the retaining plate is worn a year the tooth is liable to fall back a little. By bringing it a little too far it can be allowed to fall back. If the tooth is to be brought inward it will be retained by the pressure of the lips.

Dr. C. N. Bailey (Minneapolis) said that Dr. Angle did not claim that all the features of his system were new, but that their combination as a whole, doing away with plates and ligatures, and not removing the delicate appliance from the time it was first placed in the mouth until its work was accomplished, does constitute a new system. His jackscrews are 50 per cent. smaller than any on the market.

Dr. E. Parmley Brown (Flushing, N. Y.) said the voice of the section should be most emphatic in not letting it go out as admitted that this was in any wise *a new system*. Illustrations in the *Cosmos* for the past ten years cover every thing in the paper.

Dr. Angle wished to ask where these illustrations were to be found in the *Cosmos*, as he was not aware of its being so.

The President, Dr. Taft, reiterated the announcement that the discussion must be confined to basal principles, not mechanical details.

Dr. A. E. Baldwin (Chicago) thought it only fair to allow the essayist to explain the disputed points.

The Secretary, Dr. Dudley, reminded the members that the papers that were accepted would be published in the Transactions of the Congress, and what was said in the discussions would appear in the published discussions. As a section we had no voice in the matter of putting in or leaving out.

Dr. Farrar hoped that when his book came out it would not be said that he had stolen Dr. Angle's methods.

Dr. Talbot said that he was in the same position as Dr. Farrar. He had a book coming out, and he uses the same band, clamp, pin, etc., as Dr. Angle. The same screw is used in all machine shops. He wished to go on record as saying *now* that he has the same bands, screws, etc., as Dr. Angle. Now is the time to place these things in their proper light.

The President here accorded Dr. Angle a few moments for explanation.

Dr. Angle said that while it was very true that the principles of mechanics were all old he claimed that his application of them was new. Dr. Farrar solders his screw—Dr. Angle attaches by means of a hollow pipe. Dr. Farrar's method takes hours—his only fifteen minutes. He had examined all other appliances as far as possible, but had found them all entirely different from his own. He did not claim any originality for the jackscrew, but he did for his bands and pipes.

Dr. Storey (Dallas, Texas): Are we here to establish principles for our guidance or to establish prior claims to title of inventions? Every man should be proud to give his best to the profession, not to wrangle over who first got it out. We should say, here it is; you are welcome to it.

Adjourned.

FRIDAY, September 9, 11 A. M.

Professor Busch, Director of the Dental Institute of the Royal University of Berlin, Germany, read a paper on

THE COMPARATIVE PATHOLOGY OF THE TEETH WITH SPECIAL REFERENCE TO THE TUSK OF THE ELEPHANT

illustrated by a magnificent collection of pathological specimens of ivory.

He said that though the specimens presented were not those of human teeth they were from the dental organs of a larger mammal, so similar in the light of comparative anatomy that the relations of cause and effect could be clearly traced in the one through study of the other, where the larger scale afforded great advantages for observation. In some of the specimens there were defects like caries in nearly all respects but not connected with the pulp chamber; in some the pulp had been protected by nature's efforts, by the deposition of lime salts. In other specimens it appeared probable that some boring animal may have begun the injury, from which disintegration had spread, though this could not be positively proved. Although actual caries seldom occurs in these larger animals, alveolar abscess is comparatively frequent, and is found in all species. One specimen showed the work of destruction in the root of an elephant's tooth, then a rounding off of the edges and the cessation of the destructive operation; another a pus cavity near the pulp chamber; a third a formation of secondary dentine, nature protecting the pulp from the irritation of an alveolar abscess, the cavity of the abscess proper being close to the pulp chamber but with enough dentine to protect the pulp. Pus had evidently invaded the pulp chamber and secondary dentine had been thrown out.

Another specimen shows an abscess in the pulp itself, with a formation of secondary dentine, the pulp space being very much circumscribed and crowded, it having eventually dried up leaving the cavity empty. Another specimen had no sign outside to indicate a cavity inside of the solid dentine which must have originated in the pulp chamber, the pus gradually pressing against the sides until it forced an opening into the dentine; secondary dentine then filled up the path but left the cavity. The growth of the tusk never ceases during life. Another specimen shows an irregular development and deposits of dentine; there is a frequent tendency to mass dentine in spots like bullets—spherical bodies plainly to be seen with the naked eye, and entirely separated from ordinary dentine like a ball in a rocket, probably separated by a process of dessication.

Two other specimens show dental nodules, detached in the pulp chamber though they must have formed in the soft pulp and have been attached to the walls of the chamber; through reaction they have been severed, though one specimen shows the attachment to the wall still existing. From the position it is safe to assume that all nodular deposits have their origin at the side wall of the pulp chamber, and after reaction, separation takes place and the nodule is left loose in the soft tissues of the pulp itself.

Another specimen shows union of a fracture in the root, with a large callous and osseous union of a longitudinal fracture, the healing process being sufficient to establish reunion. In human teeth this union by callous, like other bones, sometimes takes place but very rarely. Another specimen represents the effects of external injury on dentine. In this case an iron bullet has penetrated the dentine, pierced the pulp, and lodged on the opposite side of the pulp chamber; the opening has been closed with secondary dentine which also causes a thickening on the inside. In another specimen a bullet of lead is imbedded in the dentine which did not penetrate to the pulp chamber, but there is a formation of secondary dentine at the point of entrance. It is generally supposed that the formation of secondary dentine is exclusively from pulp-function, but as the pulp was not reached in this case it was apparently formed by the alveolar membrane.

Prof. Busch introduced a very valuable little novelty in the shape ot a series of cutting cylinders of different sizes, for the quick and painless removal of moles and warts of all descriptions.

A cylinder must be selected of the exact circumference of the mole to be operated on: by quick rotation it cuts through the skin very rapidly, the centre being clipped with the scissors. A dressing to check hemorrhage is applied on cotton and left till it falls off—perhaps eight or ten days—when nothing will be felt but a scarcely perceptible white scar; a great improvement over a mole or wart. The largest size (1½ cemtim.) is as large as it is safe to use for this method. He also exhibited a bottle of specimens removed during his last course of lectures at the University of Berlin.

Dr. Wm. H. Atkinson opened the discussion of this paper.

He said he was overwhelmed with the magnitude of the presentment. The question was how to so utilize it as to obtain the greatest value. The collection of specimens represented a lifetime of observations in the shops of ivory workers—pieces discarded by them as valueless, and yet so valuable to us as the means of revealing more than we can appreciate or comprehend in the settlement of scientific problems. It is difficult for us to criticise such a presentment in the legitimate sense of criticism, and yet consciousness sees a lapse of deductions from other observations.

Not in the spirit of criticism but of inquiry, I say that in almost every attempt to show *why* instead of *how* these things are brought about we get entangled in a maze of confusion. I have been in the shops and cut the teeth and have seen that *ageing* gives the globe in the centre; the building power is minified. I know the *why* of nothing, and the *how* in such small degree that I am ashamed to attempt to unravel it, but only give what I have received till men get their own revealment: give it as foresteps of the preparatory stages by which we attain and record our scientific knowledge. I would I had the tongue of an angel, to speak from the fullness of my heart. Whether from the periphery of the pulp or from the odontoblasts it is not essential which first. In dealing with irregularities if we cut below these points we will see globules there. In complete consolidation there is a point or centre and concentric rings around. There is a lack of clean cut definition in some parts of the paper. It smells as if some of the study was done in the teeth of eels. Elephant's tusks may have a tip of enamel on the end, but it is only a cap; there is no instance where there was not an enamel organ as the forestep. I am dead against the term epithelium; it leads us astray. I would like to knock it into a cocked hat. Indifferent corpuscles—bone-plate—nerve-plate—muscle-plate—you can't differentiate; you say they are all epithelia—they are all indifferent corpuscles. This specimen is only a reminiscence of when it was an eel, before it became a whale! I developed deciduous teeth in the cetaceous stage, which were shed before I was born, but I hardly recall my sensations and observations, but something behind laid out the order of things. Some of these specimens tell a different story to me. There has been secondary dentine there, but it has melted down, decalcified; but it was not taken away; it was formed into magma and again consolidated, but does not conform to the dentinal tubules and connective tissues between. In others there was no abscess at all; there was pulp-tissue within those smooth walls. I don't say I know the causes, or *how*, but I take it for granted there was no abscess when there was no sanies, no ichor. The process going on was a minus operation of the building power.

Dr. W. C. Barrett—I thought I knew where I was when Prof. Busch was speaking, but we have been lead so far off that I don't know now.

Dr. Fredrichs (New Orleans) wanted to ask Prof. Busch to explain the formation of abscess in those cavities which are entirely enclosed. In human teeth we sometimes have such deposits of secondary dentine that the pulp is partly cut off. Sometimes when there is a globular development, a horn runs high up and we find the pulp where we did not expect it. We always associate pus with abscess. The inference I draw is that there was a mass of inclosed, encysted pus. Would like to hear from Prof. Busch what he means by what he calls abscess. Nomenclature has so much to do with a correct understanding.

Prof. Busch said that he could not positively account for all the phenomena. When there was irritation and inflammation pus forms: then secondary dentine forms a separating wall, leaving the cavity filled with pus. On first cutting teeth and opening one of these cavities the penetrating putrid odor is unmistakable. It is too imaginary to suppose there were two pulp chambers, but a separating wall was formed cutting off the pus cavity from the pulp chamber.

Dr. Fredrichs said that the function of the pulp of the elephant's tooth was different from the function of the human tooth if Prof. Busch's explanation is the true one. All will admit that pus is formed from the human pulp only after its death or destruction. He could not conceive how it could continue to perform its normal function in the production of secondary dentine to encyst the pus formed by its own death.

Dr. W. C. Barrett said that the tooth of an elephant arises from a persistent germ, its growth of elongation throughout life being similar to the growth of endogenous plants. It is true that there is no pus without inflammation at some point where microbes have access without which there can be no pus.

If an elephant's tooth or tusk is wounded at the base, in its early stage of development it may become infected by the access of microbes and pus formation follow. With the elongation of the tusk and the continued deposits of dentine from the dentinal cells, coalescence will take place and the cavity be carried along and entirely covered in, and thus eventually be found in the solid portion of the tusk. A bridge of dentine may also be formed across leaving a cavity. Under the influence of microbes, gelatinous matter and indifferent corpuscles may be melted down and reduced to a fluid state, and thus it is quite possible to com prehend the formation of a cavity within the body of dentine, containing debris.

Dr. A. E. Baldwin (Chicago). It has been asserted positively that the presence of microbes is necessary to the production of pus and no one has protested. I rise to take direct issue on that point. Though we have microscopic organisms everywhere, in the atmosphere, etc., they are not present in the formation of the pus of a felon, when it is formed beneath the periosteum. If present then they can only enter by the circulation, and if they are in the blood there is no protection to germicides. With the evidence we have I protest against the statement.

Dr. W. C. Barrett. I did not think it necessary to argue that point in the present state of Bacterial Science. They get there all the same; I don't say how.

Dr. Baldwin. I still protest. Dr. Barrett may be mistaken, like others.

Dr. A. H. Thompson (Topeka, Kansas). I would suggest that we give the precedence to foreign papers.

Dr. J. Hall Moore (Richmond, Va). It should be remembered that we have lost much valuable time in wrangling over priority in inventions and other points foreign to scientific discussions. I wish to offer as a resolution that, Whereas we are governed by the Code of Ethics of Medicine, all papers giving modes of operating, cuts, diagrams, models, be excluded.

The President Dr. Taft, said that the editor of the transactions would act in that matter.

Dr. A. E. Baldwin, said that his only object in taking a patent was to protect from shelving by the depots. When patented it would be given to the profession.

Dr. Dudley said there was nothing in the ethics of the medical profession preventing patents on surgical appliances. A motion to lay the resolution on the table was lost, and the resolution was adopted.

Adjourned.

FRIDAY, 3.15 P. M.

Dr. W. W. H. Thackston (Farmville, Va.) in the chair.

Dr. W. W. Allport (Chicago) moved that in view of the large number of valuable papers still unread, there be no more general discussions; limiting discussions to the opening appointed on the programme and reply by the author.

Dr. A. E. Baldwin hoped the resolution would not pass, as it would cut off those who might have valuable ideas to offer. He thought it better to get all the good possible out of our papers than to rapidly rush through a large number.

Dr. W. H. Atkinson seconded Dr. Baldwin's idea. There was nothing gained by the accumulation of a great mass of material.

Dr. Allport withdrew his motion.

Dr. L. D. Shepard (Boston) then read an abridged translation of a paper on

THE SIXTH YEAR MOLAR BY DR. E. ANDRIEW, PARIS, FRANCE.

Quoting from Magitot to the effect that the sixth year molar is a tooth of transition between the temporary and the permanent teeth, the second or twelfth year molar being the first real permanent tooth, arising from a bud from the sixth year molar, as the anterior permanent teeth are connected with the twenty deciduous teeth, except that the growth of the follicle of the twelfth year molar is horizontal instead of up and down as in the anterior teeth, the third molar taking the same course as the second; the first molar being the point of departure for the two others.

The first molar begins its growth in the fifteenth week of life, and is not finished until the sixth year; the second molar begins in the third month after birth; the third molar in the third year. Twenty permanent teeth originate in the germs of the deciduous teeth. The first permanent molar originates the same as the temporary teeth; the second and third molars from it like replacing teeth. The sixth-year molar originates in the epithelial band like the temporary teeth and is not truly a permanent tooth. At the age of five years a child has its twenty milk teeth. At six or six-and-a-half years comes a tooth to stay during the loss of the others. From this spring the second and third to be used during life. The canine and the bicuspids occupy the same space as the two molars, the sixth-year molar being in the centre. The pulp chamber is larger than in the other molars and the canals more slowly obliterated by dentiue. Its physiological object is to serve as a dividing line, marking the space occupied by the deciduous teeth; to maintain cohesion or articulation, during the changing of the teeth at the desired height, and to serve for masticating purposes. Its period of greatest usefulness is from its eruption until the neighboring teeth are in place, but after that it is not needed, and its retention is injurious; it is emphatically an organ of transition, and should be extracted as soon as circumstances demand it. Statistics show that seventy-four out of every one hundred decay early. Of fifty-five persons examined only two had the four first molars; eleven had three of them, and in forty-one they were all gone. Seventy-five out of a hundred lose them as a general rule. There are four special reasons for their early decay. Their density is less; the external configuration of crown is unfavorable; they are bathed in acid fluids during the displacement of the temporary teeth; the proximity of the temporary molars which are always decayed before they are lost.

The coefficient of resistance is less than in others whose density is greater. Food lodges in the interstices of the temporary teeth, which are not filled, and ferments, becoming sinks of infection, rendering the whole mouth acid. The decay of the deciduous second molars is hurtful to the sixth-year molars.

The wisdom tooth is the next earliest to decay. The lower one has but little room and is often covered with a flap of gum. The upper wisdom tooth has plenty of room but its inclination allows the lodgement of food. If the sixth-year molar is extracted it does away with many of these causes of deterioration.

If the temporary teeth are good and fall out without decay; if the sixth-year molars are of good structure and do not decay; if the jaws are large with plenty of room for all the teeth; if there is good configuration of both arches—then there is no cause for removal. But these cases are rare. The teeth are usually crowded; the temporary molars decay and cause abscesses; the sixth-year molars usually decay during the first year, with soft white decay. Then it is better to extract before the twelfth-year molars erupt. The second molar will fill half the space and the bicuspid the other half; there will be no vacant space, and room for the wisdom tooth.

If attempt is made to fill the sixth-year molar it will not hold; the enamel gives away and the filling falls out. If the pulp is exposed we cannot kill it, or you would have alveolar periostitis and fistula. It does not stand alone, and its approximal decay injures other more valuable teeth. If from bad configuration the teeth require straightening, either a bicuspid or a molar must be extracted, and the sixth-year molar can best be spared from the mouth. At the time of shedding the teeth if the permanent incisors come behind the temporary the latter should be extracted; if the lateral incisors come in obliquely the temporary canines should be prematurely sacrificed and the laterals rotated. If the first bicuspids come in the space vacated by the temporary canines they will move back when the permanent canines erupt and the temporary molar can be extracted to make room for the second bicuspids. In this way the eight anterior teeth are easily regulated. In the lower jaw it is sometimes preferable to extract the first bicuspid to make room in the back of the mouth if the sixth-year molar is good. The proper time to extract the sixth-year molar is just before the appearance of he second molar.

It is the fashion just now to say don't extract; fill and preserve every thing even if it has to be built up from the gums; but this has created havoc in many mouths, with abscesses, swelled faces, and heads tied up; so we had better go back to the extraction of the sixth-year molars.

The discussion of this paper was opened by Dr. L. D. Shepard, who said that this paper had come to us from across the waters, but if it had been presented by one of our own people it would not have been considered fit to go on record as an exemplification of modern dentistry. He was sorry to have to pronounce such serious judgment. Twenty-five and fifty years ago it was customary to extract the sixth-year molars almost universally. He himsilf had sacrificed hundreds of them, but as the results of modern practice he would formulate and promulgate this idea: With thirty-two teeth in the mouth, respect is paid to each individual tooth. No tooth on mere suspicion of violation of law should be condemned without trial by jury ; it should not be anathematized as a proved culprit without good reason. If extraction became neceesary, like amputation it should be done for a determined cause—not because it is the right ear or the left; the right eye or the left; the sixth-year molar or the bicuspid, but because the selection should be based upon the study of all the laws bearing upon the case. The extraction of a tooth as a question of expediency depends upon the lesions of the teeth, the occlusion of the jaws, their arrangement in the jaws, but the result of amputation will always be an abnormal condition; it is not physioligical but pathological. Granting that the sixth-year molar is a " tooth of transition," a method of practice based on one condition only rests on a very contracted foundation. What if it is analogous to the temporary teeth? There is no provision made for its self-extraction—exfoliation—absorption of roots. This alone is sufficient argument. It is a permanent tooth. This talk of the frequency of decay of the sixth-year molars have abont the same value as statistics of deaths from smallpox where patients are allowed to die without medicine or nursing. To find the true ratio of decay we should have the ratio of their preservation to adult life when preventitives and remedial skill are applied. The sixth-year molar is as good a tooth as the twelfth-year molar or the bicuspid if it receives the same care and is treated properly. The canine is its only superior. Hence in my practice I insist on its respectful consideration and especially its proper care at the age of six or seven years, and save them the same as any other teeth.

Dr. Paul DuBois (Professor in the Dental School at Paris) said that he did not approve of the practice of Dr. Andriew. The sixth-year molar had none of the characteristics of a deciduous tooth, though circumstances combine to make it the most feeble ; it should therefore attract our earliest attention, and be early cared for, but it should be extracted only as the last extremity. Extraction changes the articulation. In civilized races we find the jaws so narrowed that often the wisdom tooth has no room, and we should not increase that tendency and further narrow the jaws, thus interfering with mastication.

Dr. Horton (Cleveland, Ohio) considered the wholesale extraction of the sixth-year molars bad practice. In his own family he had two sons. For one he filled the sixth-year molars as soon as they began to decay, and to-day at the age of thirty-five he has his thirty-two teeth. For the second son he extracted the sixth-year molars at the age of eight or nine, but instead of giving more room for the other molars, they were crowded and the wisdom teeth were forced into the masseter muscles causing great pain. They were as handsome third molars as were ever seen, but they had to be extracted and at the age of thirty he has but twenty-eight teeth. There are many things to be considered age, sex, general condition of the mouth, probabilities as to being large enough to accommodate eight more teeth. The deciduous teeth should be filled before the age of three years if the teeth need filling to save them till the proper time for them to be lost. Where we can have full control we can save all the teeth, but too often they do not come to us util they are too far gone.

Dr. Frank Abbott (New York) said that in reference to the question whether the sixth-year molar is a permanent or a temporary tooth two points are essential. The assertion that the first molar is not as well calcified as the second or third is quite gratuitous ; the microscope, however, shows that the canaliculi are larger. Though the first molar erupts independent of a bud, the same as the temporary teeth, yet there is a great difference in the buds from them. From the temporary teeth the bud of the permanent teeth goes down and comes from under ; the bud from the first molar is horizontal for the second, and the same from the second for the third. It was intended for a permanent tooth from first to last.

The next paper read was entitled

Aphthous Stomatitis, and Its Origin, by Dr. Thomas David, Paris, France, Director of the Paris Dental School.

This disease characterized by vesicular eruption of the buccal mucous membrane, sometimes taking a diptheritic or gangrenous character, and becoming very grave or even mortal, is similar to the aphthous fever of cattle. It is generally infectious eliminating a parasitic germ, found in the watery vesicles and in the blood, but not in the milk. The *Albicum Oidium* and other micrococci have been found, but its special microbe is not known. It passes from animals to man by contact and direct inoculation. It is also transferred from man to animals. There is no doubt that Aphthous Stomititis in man is the Aphthous fever of animals and the attention of governments should be called to this fact.

Dr. Paul DuBois (Paris, France) next read a paper entitled

The Necessity of an International Inquiry into the State of the Dental System among Different Peoples.

He said that to know the character of the teeth was to know the man himself, for the teeth are an indication of his food and his life-habits, hygiene, etc. This knowledge is at the bottom of the dental system. To get the elemeuts for study a committee should be appointed by the International Congress to edit a question-book in the principal languages to which answers should be obtained by competent persons. He stated that this subject would be made prominent at the Congress to be held in Paris in 1889, and he invited all present to be present at that scientific gathering. The dentists of Paris especially would be delighted to welcome their American friends and to be enabled to extend such courtesies as they were then enjoying. He said he was especially commissioned by the dentists and the Dental School of Paris to extend this invitation to the Dental Congress of 1889, at the great Paris Exposition, probably in August. The profession in France were all united in extending this invitation. Their respect for the American profession was very great and they hoped for a large attendance.

(Dr. David apologized for extending this invitation during the Medical Congress, as it was hardly suitable to invite some to a *special feast* from which others were excluded).

Dr. John S. Marshall (Chicago) read a paper entitled

OPERATION FOR THE CURE OF A PERSISTENT NEURALGIA OF BOTH TEMPORO—MAXILLARY ARTICULATIONS AND REFLECTED PAIN IN THE RIGHT BRACHIAL PLEXUS, OF EIGHT YEARS STANDING, WITH RESULTS.

Dr. Marshall said that he did not expect to throw any light on tri-facial neuralgia, but in this case the cause was unique as far as he knew. After enumerating a long list of the usual and unusual causes of this perplexing affliction, he said that he had never known a case located as in the present instance or caused by mal-position of the parts. He knew hips and ankle joints are most liable to referred pain without local lesion, as observed in chronic myelitis, etc. It may also be due to the irritation produced by mal-position of the parts in long-continued operations on the maxillary bones, as in removal of large sections; the contraction in the formation of cicatricial tissue may draw out of place bones not reunited. The pain due to long-continued operations, formation of cicatricial tissue, aneurisms, new growths, etc., is very characteristic. The operation described in the title of the paper was undertaken on the 30th of March, 1886. The patient was a seamstress forty-two years of age. Three years previously she had undergone an operation for the removal of an Osteo Sarcoma of the inferior maxillary, extending from the first bicuspid nearly to the angle of the jaw, which had left an ugly cicatrix with mal-position of the jaw, which was carried so far back to the right that the meridian line was one inch from the centre, with one-quarter of an inch space between the ends of solid bone. The motion of the jaw produced spasmodic pains in the shoulders and arms. When the jaw was at rest she was free from pain. The mouth opened only three-quarters of an inch. She was also suffering from phagedenic perecementitis—wore a full superior denture. Had tried all remedies, aud been under treatment by noted practitioners, without obtaining relief. Concluding that the pains were due to the contraction of the parts by cicatricial tissue, which diagnosis was confirmed by Dr. Walter Hay, a distinguished surgeon, an operation was performed in the mouth, for the removal of fibrous tissue. The jaw was carried back to its proper position, and the wound filled with sterilized sponge, using the bi-chloride of mercury one-thousand solution as a mouth-wash every two hours. It was hoped that the sponge would organize, but suppuration followed and it had to be removed. The patient suffered no pain while the jaw was retained in place. When the sponge was removed, the pains returned but not extending to the arm. He was convinced that if the jaw could be held in normal position a cure could be effected. To effect this object a gold crown was placed on the right bicuspid to which was soldered a solid rod of gold one and one-eighth of an inch long; a screw was cut on the other end which was inserted in a hole drilled in the ramus. This held the jaw in its normal position and an artificial denture or bridge was inserted to fill the space and counteract the pressure on the bicuspid holding the rod. On the 17th of May an external incision was made on the line of the original cicatrix—first to try a bone graft, from the outside; second to get rid of the old tissue, and third to reduce the abnormal fullness of the right side of the face. After one or two failures from again trying sterilized sponge, with drainage by strands of floss silk, with suppuration as before, and from trying two large pieces of bone as grafts, the spring also getting displaced several times, but remaining firmly in place after the latter part of September, causing no inconveuience, the opening of the jaws increasing from three-quarters of an inch to one and one-half inches in January. On the 26th January another bone graft was made using twelve small pieces from the femur of a young rabbit in two rows across the gap. No drainage was attempted. Every piece attached; the wound healed without suppuration. Union with the ramus took place, but at the other end a gap of one-half an inch was left. Another piece was inserted to fill the space, but after fifteen days it was found there was necrosis and it was abandoned. On the 20th July the screw was displaced in yawning. The apparatus was removed and a heavy plate af Weston's metal and vulcanite inserted. The success would probably have been more complete but for the age and depressed vitality of the subject. In bone grafting there are three principles to be born in mind:

First, thorough cleanliness during and after the operation ;

Second, the graft must be small and covered with a flap of periosteum ;

Third, the graft must be taken from a young and growing subject.

If human bones could be obtained from a young child immediately after death it would be the best. The rabbit died under the anesthetics used. The bone is immersed in a one-five-hundredth solution of bichloride of mercury, at the temperature of the human body, for five minutes.

In conclusion Dr. Marshall showed casts after the conclusion of the operations, and asked Dr. Atkinson, from his own experience in grafts, to open the discussion.

Dr. Wm. H. Atkinson said that thanks were due the author for his faithful details, and the clearness with which he had laid down the principles involved in bone and sponge grafting. His results also showed the folly of the old methods of drainage of any kind. He regretted truly that there was not a whole day in which to discuss this paper. The beloved young man who had no respect for effete laws, and no fear of Madame Grundy was sure of making his mark in surgery. The only trash in the paper was the poppycock quotations in trying to define what neuralgia is. It can only be defined by an illuminated mind in off-hand speech. It may be reflex action from the sound side, but there is no neuralgia from mechanical compression. The failures indicate the immediate point for illumination. What is neuralgia? Pain in the nerve. How does it come about? The current of impact of sensation runs through the waxy, semi-fluid globules of neurine and jumps from one to the other, causing a tingling sensation of pain, though sometimes it is pleasure. If we would be Masters in Israel we must not rely on old effete teachings. The whole process of evolution is through two elemental bodies which flow together developing organisms like the parents. Embryonal shapes are made up of indifferent corpuscles—albuminous matter, through the work of epiblasts, hypoblasts, mesoblasts. The layout of all the future organs is contained within the visceral walls of the embryo. The perfect peptone is the basis of all protoplasm, feeding all the tissues till embryon is fitted to breath the air of life and fulfill the type of the parents. In the bone graft there is coaptation of tissues with so thin a line of albumen, gelatine, *glue* that the reproduced structure cannot be distinguished from the original structure. In the case described in the paper it did not take because there was not perfect cleanliness, and the graft was too large. A half-inch cube of lean beef is more readily digested than a squre foot. Because it failed once or twice he should not have given up the chase of the sponge graft. When you feel as though you wished the patient was somewhere else, or yourself elsewhere you will never have any success; but when every thing is in perfect accord you will have union by first intention.

Why was there no more pain after correct occlusion was obtained? Because the nerves were not pinched and could exercise their legitimate function. There is not time to go over the leucocytes, etc., which has been well settled by Carl Heitzman.

Dr Frank Abbott asked Why, if a strip of periosteum was raised from each end of the bone and laid under the bone-graft, it did not enter in and reproduce bone growth?

Dr, Atkinson replied, because there was not enough original coagulum to nourish it.

In reply to the question why he had not carried the operation on to complete success, Dr. Marshall replied that he found from such repeated cutting there would be only cicatricial tissue which would not unite kindly by first intention, and that he might get into serious difficulty in that way. The patient was willing to go as far as his own conscience and judgment dictated. His object in using the large graft at first was to avoid opening again. Every time he repeated the operation he had to cut it open.

Dr. Atkinson said that the great mistake had been done long before Dr. Marshall had ever seen the patient, in the removal of the maxillary bone; another blunder of the old surgeon.

Dr. Marshall's serious trouble lay in being too tender-hearted and not putting the screw in far enough to make it perfectly secure.

The following papers were read by title

ARTICULATION OF ARTIFICIAL TEETH BY DR. H. L. CRUTTINDEN, NORTHFIELD, MINN.

POWER IN DENTISTRY, BY DR. W. ST. GEORGE ELLIOTT, LONDON, ENGLAND.

PORCELAIN CROWNS, BY DR. E. C. MOORE, DETROIT, MICH.

ADJOURNED TO 8 P. M.

FRIDAY, SEPTEMBER 9, 8 P. M.

Dr. W. W. H. Thackston in the chair.

Dr. A. H. Thompson (Topeka, Kansas) read a paper entitled

DOES FUNCTION CONTROL THE EVOLUTION OF STRUCTURE.

After reference to the paper on a similar subject, read by Prof. C. N. Pierce, at Minneapolis, and noting the mechanical forces exerting an influence in the development of the different organs, he said that the evolution of function is not isolated from the development of the organs, which are modified through adaptation to their environment; thus organs may be changed, or rudimentary organs developed for the performance of functions necessitated by change of environment. The development of organs is in ratio to their employment. New agents, new appetites give new organs. Everywhere we find adjustment of man to the end, of tools to the work—of teeth to their work. The structure and adaptation of the teeth is so specialized that by them points may be identified—species differentiated. On the ability to utilize food life depends, and the masticatory organs must be adapted to the food supply. Without the possibility of adaptation to changed environments, great changes would cause extinction of life. There are many instances where we recognize extensive modification due to compulsory adaptation to food environment; not the result of accident but the response to a demand—the use for a thing precedes its growth. Teeth have developed from the simplest to the most complex, with corresponding tissues to support and protect them, in accordance with the nature of the food supply. On the other hand organs atrophy as they are disused; they are either suppressed entirely or reduced to mere rudiments. The practical lesson from this is that teeth atrophy from disuse as when artificial foods are used, and that proper education as to correct habits of mastication aud the use of proper foods will tend to restore the quality of the teeth. If the function disappears can the organ persist?

Dr. W. C. Barrett, who was appointed to open the discussion of this paper not being present Dr. W. H. Atkinson was called upon.

He charactirized the paper as a series of unsupported assumptions. Function is simply an antecedent. The mammalian lungs are prepared in all their beautiful perfection before ever any air enters it; the hydrostatic test for infanticide is infallible; if the lung sinks in water it has never been inflated; if it has been once inflated, it can never be compressed again. He said that such a paper from such an author reminded him of the man who went to the very door, smelt through the keyhole, and then swore there was no door, no lock, no keyhole there. Lamarck and others have contributed to our progress but they failed to see what life and death are. The machine is prepared to perform the work lying before it. Creation is too deep, too profound for such wretched babble, a jumble of nonsense which will pass for wisdom.

They say "temporary teeth are *exuviated*" and are as tickled as a child with a new rattle.

Education draws out; if something is not in, it can't be drawn out. Physical education is simply drawing something out of physics. Ask how it is done? It is done in us and for us, more than by us. In asking for light radiance rushes in and fills to the extent of the real asking. If we ask only to be filled with superficial breath we will get only that; if we ask for thorough inspiration we will get it if in thorough inspirational state. Ideas are formed, which are born into thought, becoming opinion—belief—knowledge.

The next paper read was from Dr. J. B. Davenport, Paris, France, entitled

### Harmony and Discord; Health and Disease; Healers and Hinderers.

He said that the life of the individual is the result of the action of all the organs of the body. All are vital, though some are more immediately essential than others. Comparing the body to an army, the medulla is like the general, who, if he surrenders paralyzes the entire force; the heart and lungs are the trusted corps; the nerves are the line of communication; the muscles the common soldiers, etc. In perfect health there is perfect performance in all the functions of the body. In perfect vigor of health all the functions are in harmonious relation. Disease of one organ affects another, and the balance is destroyed. Two parallel lines diverging ever so little will finally get very far apart. The slightest failure of the heart will soon result in death. In other organs there may be easy gradations, from mere uneasiness on, but the failure of one weakens others, which are called on to exercise vicarious functions. If treatment is opposed to natural laws it will hasten the evil result. No new organs will replace those removed by the surgeon. Man needs a varied supply of food. The dentist stands guard over the door to the digestive apparatus, and should not mar and mangle what is so well fitted to perform its work. The beginning of the process of digestion is coincident with mastication and deglutition. The saliva is a true digestive fluid and we must assume that it is essential in the perfect performance of that function. To ensure the proper supply both sides of the jaws should be equally competent to perform their functions. The loss of teeth on one side will involve excessive labor of the masticatory members on the other side. Thirty-two teeth were given to man because that is what he needs. The savages make better use of their teeth in the preparation of their food than we do; consequently the teeth of civilized men are more subject to disease. American teeth are softer than those of the Scotch and English. Civilized man makes less use of his teeth than the savage, but the latter only uses them as nature designed. Should civilized man have less teeth? Some say yes, and extract certain teeth as a rule, but with every tooth that is lost the food is less perfectly prepared for the stomach. To what point this may be carried and the human race not suffer we do not know, but to be on the safe side it is better to retain them all and exercise them properly. The size and shape of the teeth in relation to the jaws, the proper number of the teeth, the glands, vessels, etc., all are related to the architecture of the face. We may think one item unimportant but we should not assume to know or to criticize the means and ends of creative work. In war, famine, poverty, our hardships may exceed those of savage men. There are some conditions of life in which thirty-two teeth do not seem to be necessary, but they may be called into rquisition under changed conditions. Nature is not so extravagant as to provide against extractions or excisions, and there is no third denture to supply the loss of all. If teeth were ever modified by function it was when ages were as hours. Because some men have no wisdom teeth or no lateral incisors is no reason for believing that they are becoming rudimentary; they are still as necessary as ever. The loss of a tooth does not mean only a loss of masticating surface but also derangement of the remaining teeth. They are robbed of their support, and lose their function through tipping. Extraction may ruin both occlusion and mastication. There are three molars. The first one may not be as good a tooth as the others but it is needed for the support of the second and third. If nothing can be saved but the roots, they should be saved for the protection of the others. The bicuspids seem to have but little work to do, but they preserve the features and support the other teeth. Much harm has been caused by bad dentistry, opposed to natural laws. Some men have a series of set rules and endeavor to adapt each case to their rules; to deal with groups instead of individuals. Treatment should vary according to circumstances. The most important qualification of the dentist is common sense. If knowledge were as broad as the universe we might cease making mistakes.

Dr. Wm. H. Atkinson was appointed, on the programme, to open the discussion of this paper.

He said: "In proof-reading we sometimes write on the margin *stet.* That is all I have to say."

Dr. Cunningham (England) whose papers had not arrived when Dr. Cravens read his paper, and who had been accorded the privilege of reopening the subject for Dr. Cravens' final reply, now gave a series of statistics in reference to the treatment of pulpless teeth by his method of "immediate root-filling."

His practice is to remove all softened dentine from the pulp chamber, but not to open the root canals; wipe out with carbolic acid on cotton; apply arsenious acid to the opening of the root canal, covering it with a disk of paper dipped in carbolic acid; fill over this with oxychloride, and complete the filling with the material thought best. His success had been such that he confidently recommended this method. He admitted, howerer, that as his practice was in a university town he never saw many of his patients a second time, but they had the opportunity of returning if there had been any subsequent suffering, which was not the case. He thought if there had been failures he would have heard of them. University students cannot pay the frequent visits to the dentist necessary under the old system of repeated dressings, and will let their teeth go unfilled if told to come back again. Some succeed perfectly in filling all root canals, and some don't; himself being in the latter category. For these two reasons he had adopted the above plan. Recently traveling on the Continent he visited the German school at Liepsic. He found there were several who wanted to communicate to him something they considered "the newest and the best," and they all had the same thing—immediate root-filling. Dr. Smith Dodge was the first one to give it that name. His own experience in that method dates back to 1883. As a case in point he was on one occasion about to operate for a friend. He had put on the rubber dam and removed an old filling when the patient was summoned away by an urgent message, and the tooth was left open. The next day he had about the brightest face ever seen. If the intended filling had been put in he would have said he had obstructed the passage-way of the germs! He uses eucalyptus and iodine; creosote, eucalyptus and iodine, and in some cases oil of cloves. (Then follows a long list of percentages of successes and failures by the use of different dressings and methods). In applying the arsenic to the root canals he wraps a shred of cotton on a nerve bristle and dips it in the oil of cloves and takes up a very slight portion of arsenic carrying it as near to the apex as he deems safe, but this must be done with great caution. A one per cent. solution of arsenious acid in glycerine, agitating it in a water-bath, gives a safe preparation for this purpose; also a two per cent. solution in alcohol and oil of cloves. He speaks of many cases of what he calls "inherent spontaneous curability"—that is where there has been an abscess but we find cicatricial tissue and no present abscess. If we use the nerve drill and remove as much as possible we may anticipate strife between the diseased portion and the sound bone at the end. A slight degree of subsequent inflammation is an aid rather than an obstacle in the removal of old inflammatory products; if the pain is severe it may be combatted by local applications and quinine internally. This diminishes inflammatory action, though *how* we don't know, but he vouches for its benefit and the subsequent gratitude of the patient. The inflammatory process is so slight as often to pass unnotieed. In many cases the matter seems to dry up or form a caseous mass. If the fistula persists, resort to heroic treatment; burr out and treat with sulphuric acid, followed by resorcin. It will heal more rapidly than if treated from the interior. Rhizodontrophy he condemns. Drainage tubes unnecessary. He replants only in very rare cases. The pathology of his method may be subject to debate but he pleads the silent eloquence of facts, and is content with the manifest advantages of his method over the old long-continued dressings. He has fewer extractions, fewer swollen faces, fewer abscesses, less pain, and consumes less time for both himself and the patient, in many cases consuming less than half an hour. He leaves broken drills in with impunity and has yet to find one case where they have caused trouble. He asks for a fair discussion.

Dr. Kirk (Indiana) said that seventeen years ago a patient came in to have a tooth filled, insisting on its being done immediately, as he was just starting for Kansas, and it must be done. There was a dead nerve, but under the circumstances he filled it with oxychloride of zinc, and dismissed the patient, hoping he would never meet him again. Three months later the man came back, and the doctor dodged him carefully for three weeks, until the man found him in his office when he came to thank him for the service rendered, the tooth having given perfect satisfaction except for the first three days when it gave him—he would not say what. On another occasion he put in, for a boot-black, a gold filling over oxychloride of zinc, with a badly swollen face, with no bad results. He now frequently fills with chloride of zinc and gutta percha, without any treatment, and although an occasional swelled face follows he has less subsequent trouble than by any other method, or than any one he knows who follows the old mode of dressings. He fills the apex with gutta percha, washes out with alcohol and dries with hot air. Keep drills out of nerve canals.

Dr. Ames (Chicago) gave the method he has used for five years. He believes in thorough disinfection and removal of contents, depending on the accessibility and size of the canals. He decomposes the contents of canals by electrolysis, flooding with an acidulated fluid, he passes a platinum probe to the apex, reaching the smallest point; the decomposition liberates nascent oxygen which combines with sulphuretted hydrogen and acts as a disinfectant.

Dr. Conrad (St. Louis) had cliniced on immediate root-filling. Had gained confidence in the method by oft-repeated success.

Dr. A. E. Baldwin (Chicago) had not heard all of Dr. Cravens' paper, but was an advocate of immediate root-filling. If there was a blind abscess would fill after thoroughly drying to the apex. If there was a fistula would treat from the outside. He feared micro-organisms less than Dr. Barrett, and would take exception to the statement that there would be no pus without their presence. He would affirm that more harm than good was done by medicinal agents. He had been greatly interested in the statements made by the gentleman from England (Dr. Cunningham) and had great respect for honest opinion. Ridicule is not discussion; sneering remarks reflect more on the speaker than on the author and are non-professional.

Dr. Sitherwood was gratified by the paper of the gentleman from England. It had been his practice for six years to fill at a single sitting. He believed that success depended on thorough cleansing. He has comparatively few failures, and believes that the majority of dentists now fill at one sitting.

Dr. Storey (Dallas, Texas) said that if everything was thoroughly cleaned out, and nothing left to be treated it made very little difference what you treated with. He used no drills; got every thing out that he could, but did not want to get any thing in. If he has any failures they all go to "the other fellows;" they don't come back to him, and he never hears of them.

Dr. Stack (Dublin) said that he had but little to add. He found that in America the people were educated in the proper care of the teeth; it was comparatively rare here to find teeth so neglected as they are when they come under the hands of the English or the Irish dentist. He could not say he never had a swelled face. Dr. Cunningham had presented a strong array of facts and statistics, but he did not think American dentists were the ones to discuss their methods, as circumstances were so different that their difficulties could not be understood. The people of America had been so much better taught in the care of the teeth that the American dentist has comparatively no difficulties to contend with.

Dr. Cravens thanked the gentlman for reopening the subject and Dr. Fillibrown for the fair, gentlemanly and scholarly manner in which he had discussed his paper. There were some points that demanded a reply. First, as to the criticism of his practice of *waiting* when a tooth presented was too sore to admit of operating; he often found teeth so sore as to render manipulation impracticable. He gave patients instructions for home-treatment to reduce inflammation and modify pain. It did not necessitate long waiting. He was not afraid of the bugaboo of septic conditions corked up. Objection was made to his term "apical space," but it was a term so fixed in dental nomenclature that it was easier to use it than to displace it. It was said that he failed to mention "impenetrable pulp canals," but he had not attempted every possible case, which would not be within the limits of a paper. Roots were often too slender to be filled with solids, but they could be explored, and could be stopped with shellac on a hog's bristle, permanently left in, using the end which came out of the skin for the advance-end, which would not pass beyond the point of constriction. Dr. Fillibrown had criticised his method of using phosphate of lime for filling roots of deciduous teeth as being in contradiction of his dictum—" use no medicines, etc. He used this expression in the same sense as the cook who uses salt and yeast-powder in biscuits but who would justly disclaim with indignation the imputation of putting " medicine" in the food. It is a powder which is easily shifted to meet the demands of absorption. He only asked a fair trial of his method, with the warning that it requires courage to pursue any but the beaten path.

Dr. Wm. H. Atkinson said that when a tooth was too tender for manipulation, if a loop of floss silk is passed around it and an instrument passed through it to draw it tight with instructions to the patient to tighten it until he feels no pain, you can without any trouble " cut it out, clean it, fill it, kiss her good-bye—amen!"

Dr. J. Rollo Knapp (New Orleans) moved the appointment of a committee of two to draw up suitable resolutions of appreciation of the services of Dr. N. S. Davis in favor of dentistry.

Passed unanimously.

Dr. Dwinelle hoped the section would not adjourn until a similar vote of thanks had been passed to our worthy President, Dr. J. Taft, for the able manner in which he had presided over the deliberations of the meeting, overcoming the many difficulties in a most masterly way.

Passed unanimously with hearty applause.

President Taft said that he fully appreciated this expression, but that he felt his success had fallen far short of what it might have been in other hands. We were now nearing the close of the sessions of the Section of Dental and Oral Surgery of the Ninth International Medical Congress. During the past two years a variety of opinions had been held on the subject, but it had finally culminated in a reasonable success. He disclaimed all personal credit for the results which were due to the united efforts of all in attendance. Thanks are due to all for what has been accomplished, and for the manner in which it has been accomplished. But, what shall be the result? There has never before been such a gathering of dentists, assembled in harmony, working for the accomplishment of a given purpose.

Great fruits must grow from the seed sown at this meeting. Will it stop here? By no means. It will go out into all the world, and reach our professional brethren wherever they may be on the face of the broad earth. Each one of us will carry with him what will enlighten others. No man can estimate the good that will come from it to those who may be under our charge. The other Sections of the Congress have known but little of what we have been doing, but the general impression is that this section has been one of the best of the Congress; it has been unusually well attended; even on this, the last day, there is a record of 427 in attendance, and nearly all members of our profession. At this hour—10.30 P. M. there are nearly as many here as at any preceding time; this speaks strongly. It will not stop with us, and those under our hands as patients.

What we have done will be published in the transactions, and the members of the other sections will learn of the work here, as we will learn what the physicians have done. It will be a valuable record for reference at any future time. It is impossible to estimate the ultimate results of the work done here this week. In its social aspects and features links have been forged that will not soon be broken; the bonds of sympathy and good fellowship have been strengthened by personal intercourse. As we part pleasant memories will go with us.

It now remains only to announce that the Dental and Oral Section of the Ninth International Medical Congress—Dr. Cunningham interrupting the President at this point said: We cannot adjourn without thanking the secretaries for the faithful performance of their duties; as also the Executive Committee, for they have done wonders. We shall also look forward to meeting again. I am sure that the other members of the profession, from England, unite with me in the expression of gratification at the success of this section which has been one of the most distinguished and the most largely attended of all the Congress. I move a special vote of thanks to Dr. Dudley.

Carried with a rising vote.

The President then concluded his announcement to the final.

Adjourned, and the Dental and Oral Section of the Ninth International Medical Congress became a thing of the past.

